# Clinical characteristics and prognostic differences of children with acute bronchitis across different age groups

**DOI:** 10.3389/fmed.2026.1922135

**Published:** 2026-08-17

**Authors:** Chao Yan, Qiang Gao, Jinliang Ya

**Affiliations:** 1 Department of Pediatrics, Linquan County People’s Hospital, Fuyang, Anhui, China; 2 Department of Laboratory Medicine, Linquan County People’s Hospital, Fuyang, Anhui, China

**Keywords:** acute bronchitis, anemia, children, chronic cough, premature birth

## Abstract

**Objective:**

This study aimed to analyze the clinical characteristics of children with acute bronchitis in different age groups and to identify the risk factors for the development of chronic cough in these children.

**Methods:**

Clinical data of 290 children hospitalized for acute bronchitis at Linquan County People’s Hospital from August 2022 to September 2024 were retrospectively collected. The children were categorized into three age groups: <1 year, ≥1 year and <3 years, and ≥3 years. Data collected included gender, age, home address, gestational age, birth weight, parental smoking status, symptoms such as stuffy nose, sputum, fever, peak temperature, fever duration, hospitalization duration, anemia, white blood cell count (WBC), C-reactive protein, neutrophils (Neu), and lymphocytes. The children were further divided into a good prognosis group (*n* = 256) and a poor prognosis group (*n* = 34) based on the presence of chronic cough. Univariate and multivariate logistic regression analyzes were performed on all variables, and a nomogram model was constructed. The predictive ability of the model was evaluated using ROC curves.

**Results:**

Significant differences were observed among age groups in WBC and Neu levels and in the proportions of children with stuffy nose, sputum, and fever. Among the 290 children, 34 (11.7%) developed chronic cough. In multivariable logistic regression analysis, gestational age (adjusted OR [aOR], 0.385; 95% CI, 0.263–0.563), parental smoking (aOR, 2.479; 95% CI, 1.104–5.569), and anemia (aOR, 4.734; 95% CI, 1.539–14.563) were independently associated with chronic cough. The nomogram showed good discrimination, with an AUC of 0.819 (95% CI, 0.735–0.903).

**Conclusion:**

The clinical characteristics and symptoms of children with acute bronchitis differ by age. Gestational age, parental smoking, and anemia were independently associated with chronic cough following acute bronchitis. The nomogram may help identify children at higher risk of chronic cough, although external validation is warranted.

## 1 Introduction

Lower respiratory tract infections have become the leading infectious cause of death worldwide, with children facing a higher risk of mortality compared to adults, which make them more prone to severe respiratory complications (1). Acute bronchitis, a common lower respiratory tract infection, is primarily associated with viral infections. Its hallmark symptom is a persistent cough lasting from a few days to weeks, often accompanied by sputum production (2).

Previous studies have shown that acute bronchitis, as a self-limiting disease, typically has no long-term effects and usually only requires non-pharmacologic or symptomatic treatments (2). Cold symptoms such as nasal congestion and fever generally improve within a few days, with the cough resolving within weeks. However, it is important to note that some children may still require hospitalization due to complications or severe symptoms (3–6).

Individuals of different ages exhibit variations in physiological, immunological, and metabolic factors, leading to differences in clinical manifestations, disease severity, and therapeutic responses. These variations are also evident in children across different age groups. Previous studies have analyzed the clinical characteristics of children with COVID-19 and *Mycoplasma pneumoniae* pneumonia across age groups, highlighting age-related differences in disease presentation and outcomes (7, 8). However, the clinical characteristics of children with acute bronchitis across different age groups remain unknown.

Post-infectious cough (PIC) is a common complication following respiratory infections, typically characterized by the persistence of a dry, irritating cough or a cough with minimal mucus production, even after the primary infection has resolved (9). For most patients, PIC is a subacute cough that lasts for a few weeks and eventually resolves (2, 9). However, it is important to note that some individuals may develop a chronic cough that persists longer, significantly impacting their daily activities and quality of life (9). There is no specific clinical treatment for post-infectious cough (PIC), and symptomatic relief is mainly achieved through the use of cough suppressants or antihistamines. However, these medications often come with unavoidable side effects (9, 10). Therefore, identifying the factors that influence the development of PIC is crucial for the early detection and management of at-risk patients. Studies have examined the factors contributing to chronic cough following COVID-19 infection, providing insights into potential risk factors for PIC development (11). As an infectious disease, acute bronchitis can also lead to persistent cough. However, to our knowledge, few studies have specifically examined chronic cough following acute bronchitis in children, and the risk factors for its development remain unclear.

In this study, we conducted a retrospective study to compare the clinical characteristics of children with acute bronchitis across different age groups. In addition, we will examine the risk factors associated with poor prognosis, specifically focusing on the development of chronic cough. This study aims to provide a theoretical foundation for the effective clinical management of children with acute bronchitis.

## 2 Methods

### 2.1 Study design and setting

In this study, we retrospectively collected the clinical data of 290 children admitted for acute bronchitis from August 2022 to September 2024 in the Department of Pediatrics of Linquan County People’s Hospital. The inclusion criteria were children aged under 12 years who were diagnosed with acute bronchitis and had complete clinical data available for analysis. The clinical records of included children were reviewed through November 2024 to assess the development of chronic cough. Children with cognitive dysfunction or severe organ damage were excluded from the study. The study protocol was approved by the Ethics Committee of Linquan County People’s Hospital. The diagnosis of acute bronchitis was established by experienced pediatricians based on clinical manifestations and physical examinations. The inclusion criteria required children to present with an acute onset of cough, with or without sputum production, and the presence of dry or moist rales upon auscultation. To ensure diagnostic accuracy and exclude other respiratory conditions, the following steps were taken: Exclusion of Pneumonia: Pneumonia was excluded based on the absence of typical clinical signs (such as tachypnea and chest indrawing) and, when clinically indicated, a normal chest radiograph showing no evidence of pulmonary infiltrates. Exclusion of Other Conditions: Children with a prior diagnosis of asthma, bronchiolitis, or pertussis were excluded. Furthermore, patients presenting with symptoms characteristic of these conditions, such as paroxysmal coughing with a whoop (suggestive of pertussis) or diffuse wheezing (suggestive of asthma or bronchiolitis), were not included in the study.

### 2.2 Clinical data collecting

Based on the age of the children, they were categorized into three groups: Group A (<1 year, *n* = 43), Group B (≥1 year and <3 years, *n* = 90), and Group C (≥3 years, *n* = 157). These age boundaries were selected to reflect clinically relevant differences in respiratory development, pulmonary function, and immune maturation during early childhood. Using the complete records of each child up to November 2024, all participants were assessed for the persistence of cough for more than 4 weeks after the diagnosis of acute bronchitis. The children were then classified into two groups based on the presence or absence of chronic cough: the good prognosis group (*n* = 256) and the poor prognosis group (*n* = 34; Figure S1). Chronic cough was defined as a cough lasting longer than 4 weeks in children (12).

We collected data on various clinical and demographic factors, including gender, age, home address, gestational age, birth weight, parental smoking status, presence of stuffy nose, sputum production, fever, peak temperature in febrile children, duration of fever, length of hospitalization, anemia status, and laboratory values such as white blood cell count (WBC), C-reactive protein (CRP), neutrophils (Neu), and lymphocytes (Lym) from all patients. This comprehensive dataset allowed for a detailed analysis of the clinical characteristics and prognostic factors associated with acute bronchitis in children. Only children with complete clinical data were included in the analysis; therefore, no missing data were present in the final dataset and no imputation was performed.

In order to facilitate statistical analysis, we organized the data as follows: children were classified as urban or non-urban residents based on their home address, and as children of smoking or non-smoking parents based on the smoking status of first-degree relatives. Symptoms such as nasal congestion, sputum production, and fever were used to differentiate children by the presence or absence of these symptoms. Anemia was defined according to age-specific hemoglobin thresholds recommended by the World Health Organization (WHO). Specifically, anemia was defined as a hemoglobin concentration <110 g/L for children aged 6 months to 5 years and <115 g/L for children aged 5–11 years, as applicable. Blood indices (WBC, CRP, Neu, Lym, etc.) were categorized as elevated or decreased, as they vary significantly across age groups and are not directly comparable (13). CRP levels ≥10 mg/L of the hospital laboratory were considered elevated. No additional treatment was provided based on gestational age, birth weight, temperature, fever duration, or hospitalization duration.

### 2.3 Statistical analysis

We used SPSS software version 26.0 to statistically analyze the clinical data of the patients. We first conducted the normality test for the measurement data. Measurement data meeting normal distribution were expressed as *x̄* ± *s*, and One-Way ANOVA was used for comparison among the three groups. If the measurement data did not meet normal distribution, they were expressed as median, quartile [*M* (*Q*_1_, *Q*_3_)], and Kruskal-Wallis test was used for comparison among the three groups. Bonferroni-Dunn test was used for further multiple comparisons. Count data were described as number and percentage and were statistically analyzed by *χ*^2^ test. Subsequent multiple comparisons were made by Bonferroni correction. *p* < 0.05 indicated a statistically significant difference. Subsequently, univariate and multivariate logistic regression analyzes were performed to identify significant factors. Variables with *p* < 0.05 in univariate analyzes were included as candidate predictors in the multivariable logistic regression model. A backward stepwise selection procedure was used to identify independent predictors of chronic cough. A nomogram prediction model for poor prognosis in children was constructed. The performance of the nomogram model was then evaluated using ROC and calibration curves.

## 3 Results

### 3.1 Baseline characteristics of children with acute bronchitis across three age groups

The baseline characteristics of children with acute bronchitis in the three age groups. Were shown in Table 1. The results showed that there were no significant differences among the three groups in terms of gender, age, birth weight, gestational age, home address and parents’ smoking status (*p* > 0.05).

**Table 1:** Baseline characteristics of patients in three age groups.

Variables	Group A (*n* = 43)	Group B (*n* = 90)	Group C (*n* = 157)	Statistic	*p*
Age, months, *M* (*Q*_1_, *Q*_3_)	6.0 (3.0, 9.0)	18.0 (14.0, 24.0)	48.0 (38.0, 72.0)	*H =* 245.680	<0.001
Gender, *n* (%)				*χ*^2^ = 0.764	0.682
Male	24 (55.81)	47 (52.22)	91 (57.96)		
Female	19 (44.19)	43 (47.78)	66 (42.04)		
Birthweight, *g*, *x̄* ± *s*	3,478.14 ± 337.10	3,450.59 ± 358.19	3,437.65 ± 363.01	*F* = 0.220	0.802
Gestational age, week, *M* (*Q*_1_, *Q*_3_)	38.00 (37.00, 40.00)	38.00 (37.25, 39.00)	38.00 (38.00, 39.00)	*χ*^2^ = 2.802^#^	0.246
Urban resident, *n* (%)				*χ*^2^ = 4.992	0.082
No	20 (46.51)	28 (31.11)	45 (28.66)		
Yes	23 (53.49)	62 (68.89)	112 (71.34)		
Parental smoking, *n* (%)				*χ*^2^ = 1.476	0.478
No	25 (58.14)	57 (63.33)	87 (55.41)		
Yes	18 (41.86)	33 (36.67)	70 (44.59)		

*F*: ANOVA; ^#^Kruskal-Waills test; *χ*^2^: Chi-square test.

*M*: Median; *Q*_1_: 1st Quartile; Q_3_: 3rd Quartile.

### 3.2 Laboratory indices of children with acute bronchitis in three age groups

The results of laboratory tests were shown in Table 2. The results showed that there was no significant difference in anemia, CRP and Lym among the three children groups (all *p* > 0.05). However, significant differences in WBC (*p* = 0.021) and Neu (*p* = 0.021) were observed between the two groups. By further multiple comparisons, we found that the percentage of children with reduced WBC in Group C was significantly lower than that in Group A (*p* < 0.05). The proportion of increased Neu was significantly higher in Group C than that in Group A (*p* < 0.05).

**Table 2:** Laboratory results in patients in three age groups.

Variables	Group A (*n* = 43)	Group B (*n* = 90)	Group C (*n* = 157)	Statistic	*p*
Anemia, *n* (%)				*χ*^2^ = 3.918	0.141
No	37 (86.05)	85 (94.44)	148 (94.27)		
Yes	6 (13.95)	5 (5.56)	9 (5.73)		
WBC, *n* (%)				*χ*^2^ = 7.681	**0.021**
Not decreased	33 (76.74)	82 (91.11)	143 (91.08)^a^		
Decreased	10 (23.26)	8 (8.89)	14 (8.92)		
CRP, *n* (%)				*χ*^2^ = 3.696	0.158
Not increased	28 (65.12)	72 (80.00)	121 (77.07)		
Increased	15 (34.88)	18 (20.00)	36 (22.93)		
Neu, *n* (%)				*χ*^2^ = 7.759	**0.021**
Not increased	28 (65.12)	44 (48.89)	65 (41.40)^a^		
Increased	15 (34.88)	46 (51.11)	92 (58.60)		
Lym, *n* (%)				*χ*^2^ = 2.597	0.273
Not decreased	28 (65.12)	68 (75.56)	121 (77.07)		
Decreased	15 (34.88)	22 (24.44)	36 (22.93)		

*χ*^2^: Chi-square test; ^a^Bonferroni correction.

^a^*p* < 0.05 compared to Group A.

Bold values indicate statistically significant differences.

### 3.3 Symptoms and prognosis of children with acute bronchitis in three age groups

The symptoms and prognosis of the children in the three groups were shown in Table 3. The results showed that there was a significant difference on the nasal congestion (*p* = 0.008) and sputum (*p* = 0.007) among the three groups. Compared to Group A, the proportion of nasal congestion was significantly lower in both of the other two groups, and the proportion of sputum production was also lower in Group B children (*p* < 0.05).

**Table 3:** Symptoms and prognosis of patients of three age groups.

Variables	Group A (*n* = 43)	Group B (*n* = 90)	Group C (*n* = 157)	Statistic	*p*
Stuffy nose, *n* (%)				*χ*^2^ = 9.732	**0.008**
No	25 (58.14)	74 (82.22)^a^	122 (77.71)^a^		
Yes	18 (41.86)	16 (17.78)	35 (22.29)		
Sputum, *n* (%)				*χ*^2^ = 9.974	**0.007**
No	4 (9.30)	30 (33.33)^a^	34 (21.66)		
Yes	39 (90.70)	60 (66.67)	123 (78.34)		
Fever, *n* (%)				*χ*^2^ = 10.724	**0.005**
No	18 (41.86)	47 (52.22)	49 (31.21)^b^		
Yes	25 (58.14)	43 (47.78)	108 (68.79)		
Peak temperature, °C, *M* (*Q*_1_, *Q*_3_)	38.70 (38.40, 39.30)	38.50 (38.20, 39.10)	39.20 (38.60, 39.70)^c,d^	*χ*^2^ = 21.783^#^	**<0.001**
Fever time, *d*, *M* (*Q*_1_, *Q*_3_)	2.00 (2.00, 3.00)	3.00 (2.00, 4.00)	3.00 (3.00, 4.00)^c^	*χ*^2^ = 7.678^#^	**0.022**
Hospital time, *d*, *M* (*Q*_1_, *Q*_3_)	4.00 (3.00, 4.50)	4.00 (3.00, 5.00)	4.00 (3.00, 6.00)	*χ*^2^ = 6.925^#^	**0.031**
Prognosis, *n* (%)				*χ*^2^ = 0.249	0.883
Good	37 (86.05)	80 (88.89)	139 (88.54)		
Poor	6 (13.95)	10 (11.11)	18 (11.46)		

^#^Kruskal-Waills test; *χ*^2^: Chi-square test; ^a,b^Bonferroni correction; ^c,d^Bonferroni-Dunn test.

^a,c^*p* < 0.05 compared to Group A; ^b,d^*p* < 0.05 compared to Group B.

*M*: Median; *Q*_1_: 1st Quartile; *Q*_3_: 3rd Quartile.

Bold values indicate statistically significant differences.

The proportion of fever (*p* = 0.005), peak temperature (*p* < 0.001) and duration of fever (*p* = 0.022) were significantly different among the three groups. The multiple comparison results revealed that a significantly higher proportion of children in Group C experienced fever compared to Group B, and the duration of fever was significantly longer in Group C than in Group A. Additionally, the peak body temperature was significantly higher in Group C children compared to both Group A and Group B (*p* < 0.05). Furthermore, there was a significant difference in the length of hospitalization among the three groups (*p* < 0.05), although no significant differences were observed in further multiple comparisons (*p* > 0.05). Finally, when prolonged cough was used as an indicator of prognosis, no significant differences in prognosis were found among the groups (*p* > 0.05).

### 3.4 Univariate logistic regression analysis of patients’ prognosis

Among the 290 children, 34 presented with chronic cough, while 256 did not exhibit this symptom. Based on this, the children were categorized into two groups: the good prognosis group and the poor prognosis group.

First, we used the prognosis of the children as the dependent variable and all other clinical and demographic data as independent variables. The independent variables included gender, age, residency, parental smoking status, sputum production, fever, nasal congestion, anemia, and changes in laboratory values (WBC, CRP, Neu, Lym). Continuous variables such as gestational age, birth weight, and temperature were treated as continuous data. Univariate logistic regression analysis was then performed. As shown in Table 4, gestational age (*p* < 0.001), parental smoking status (*p* = 0.014), and anemia (*p* = 0.002) were significantly associated with differences in the prognosis of acute bronchitis.

**Table 4:** Univariate logistic regression analysis of patient prognosis.

Variables	*β*	SE	*p*	OR (95% CI)
Gender				
Male				Reference
Female	−0.418	0.380	0.271	0.658 (0.312–1.387)
Age				
<1 year				Reference
≥1 year, <3 year	−0.260	0.553	0.638	0.771 (0.261–2.280)
≥3 year	−0.225	0.506	0.657	0.799 (0.296–2.155)
Gestational age	−0.949	0.186	**<0.001**	0.387 (0.269–0.557)
Birthweight	−0.000	0.001	0.792	1.000 (0.999–1.001)
Urban resident				
No				Reference
Yes	−0.309	0.378	0.414	0.734 (0.350–1.540)
Parental smoking				
No				Reference
Yes	0.924	0.375	**0.014**	2.520 (1.207–5.260)
Stuffy nose				
No				Reference
Yes	−0.421	0.473	0.373	0.656 (0.260–1.658)
Sputum				
No				Reference
Yes	−0.005	0.430	0.991	0.995 (0.428–2.313)
Fever				
No				Reference
Yes	−0.494	0.366	0.177	0.610 (0.298–1.251)
Peak temperature	0.312	0.359	0.385	1.366 (0.676–2.760)
Fever time	−0.182	0.206	0.376	0.833 (0.557–1.248)
Hospital time	0.033	0.120	0.784	1.034 (0.816–1.308)
Anemia				
No				Reference
Yes	1.578	0.511	**0.002**	4.846 (1.781–13.189)
WBC				
Not decreased				Reference
Decreased	0.380	0.525	0.469	1.462 (0.522–4.094)
CRP				
Not increased				Reference
Increased	0.162	0.416	0.697	1.176 (0.521–2.657)
Neu				
Not increased				Reference
Increased	−0.529	0.370	0.153	0.589 (0.285–1.218)
Lym				
Not decreased				Reference
Decreased	0.403	0.395	0.307	1.496 (0.691–3.243)

OR: Odds ratio; CI: Confidence interval.

Bold values indicate statistically significant differences.

### 3.5 Multivariate logistic regression analysis of prognosis factors for chronic cough

In this section, we further investigated the independent risk factors for the development of chronic cough in children with acute bronchitis by including the variables with *p* < 0.05 from the univariate logistic regression analysis. These variables were then used as independent variables in the multivariate logistic regression analysis. As shown in Table 5, lower gestational age (*p* < 0.001), parental smoking (*p* = 0.028), and anemia in children (*p* = 0.007) were identified as independent risk factors for the development of chronic cough in children with acute bronchitis.

**Table 5:** Multivariate logistic regression analysis of patient prognosis.

Variables	*β*	SE	*p*	OR (95% CI)
Gestational age	−0.955	0.193	**<0.001**	0.385 (0.263–0.563)
Smoke				
No				Reference
Yes	0.908	0.413	**0.028**	2.479 (1.104–5.569)
Anemia				
No				Reference
Yes	1.555	0.573	**0.007**	4.734 (1.539–14.563)

Bold values indicate statistically significant differences.

### 3.6 Construction of the nomogram prediction model

**Figure 1:**
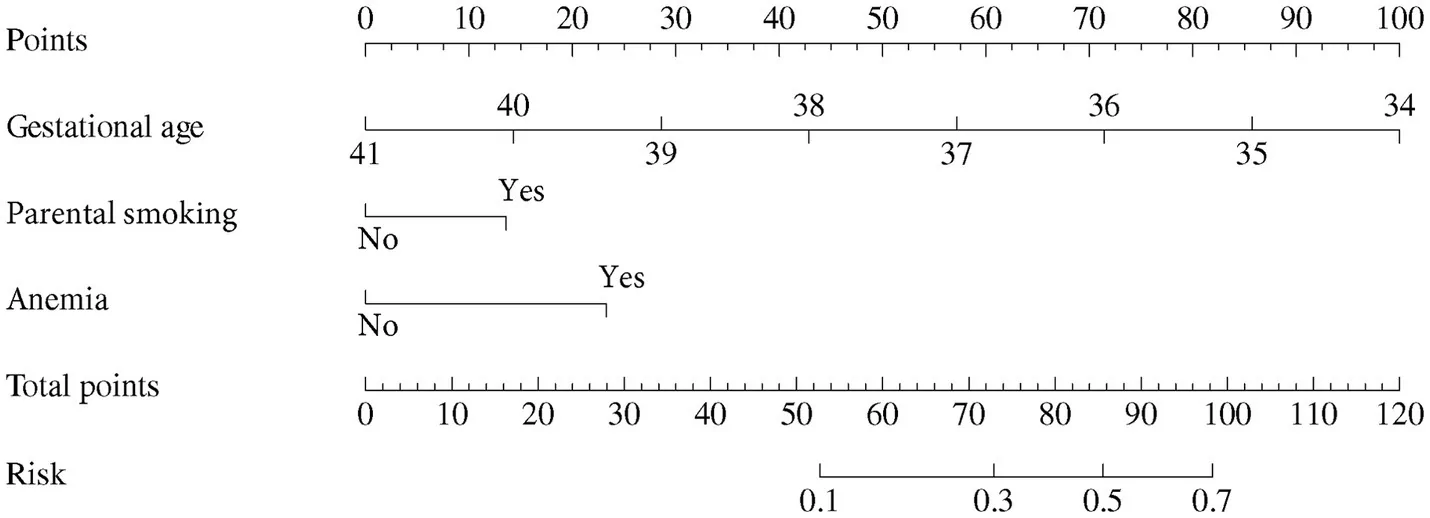
Nomogram for predicting the probability of chronic cough following acute bronchitis in children based on gestational age, parental smoking, and anemia.

Following the identification of independent risk factors for prognostic chronic cough in children with acute bronchitis (lower gestational age, parental smoking, and anemia) through multivariate logistic regression analysis, we constructed a nomogram prediction model, as shown in Figure 1. Each factor was assigned a score based on its relative influence on the dependent variable in the nomogram. The total score, derived from the sum of individual factor scores, was used to predict the risk of chronic cough in children with acute bronchitis, providing a quantitative approach to assess prognosis.

### 3.7 Evaluation of the nomogram prediction model

**Figure 2:**
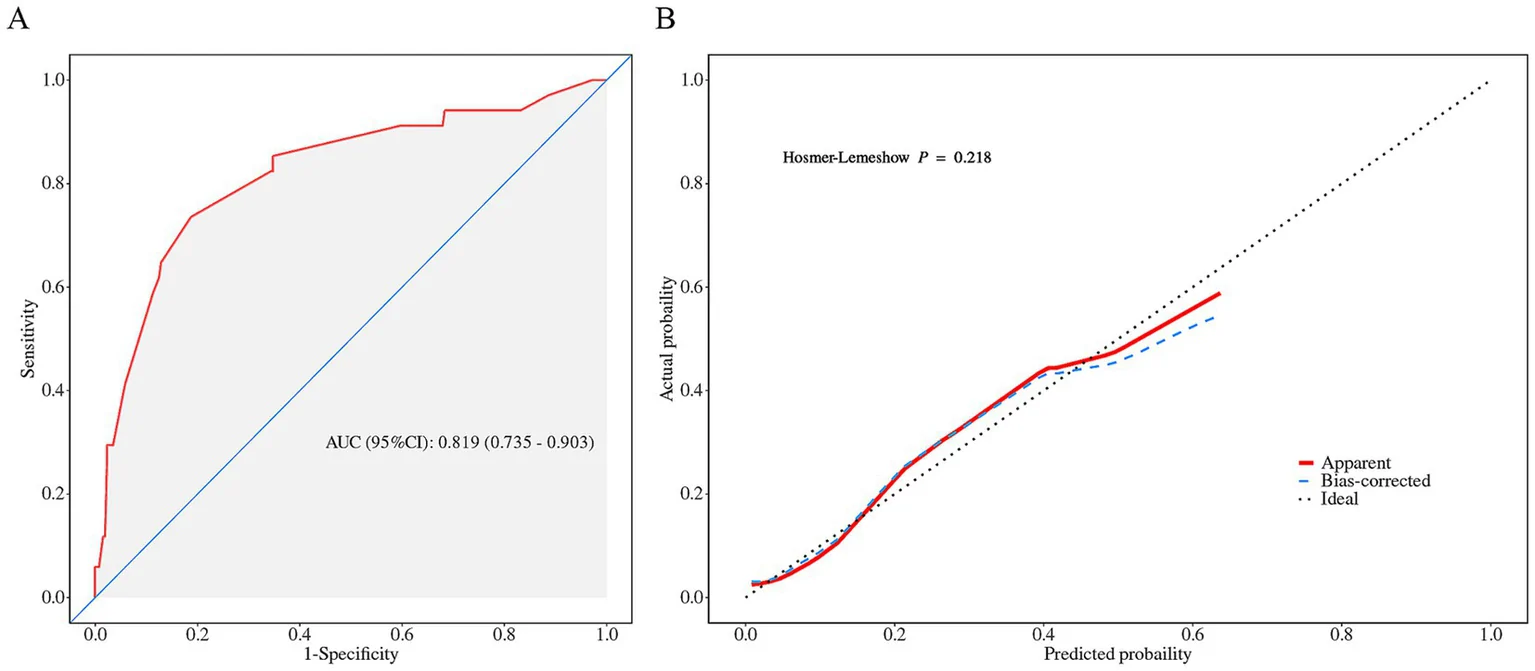
Discrimination and calibration of the nomogram model for predicting chronic cough following acute bronchitis in children. **(A)** Receiver operating characteristic (ROC) curve of the nomogram model. **(B)** Calibration curve of the nomogram model.

**Figure 3:**
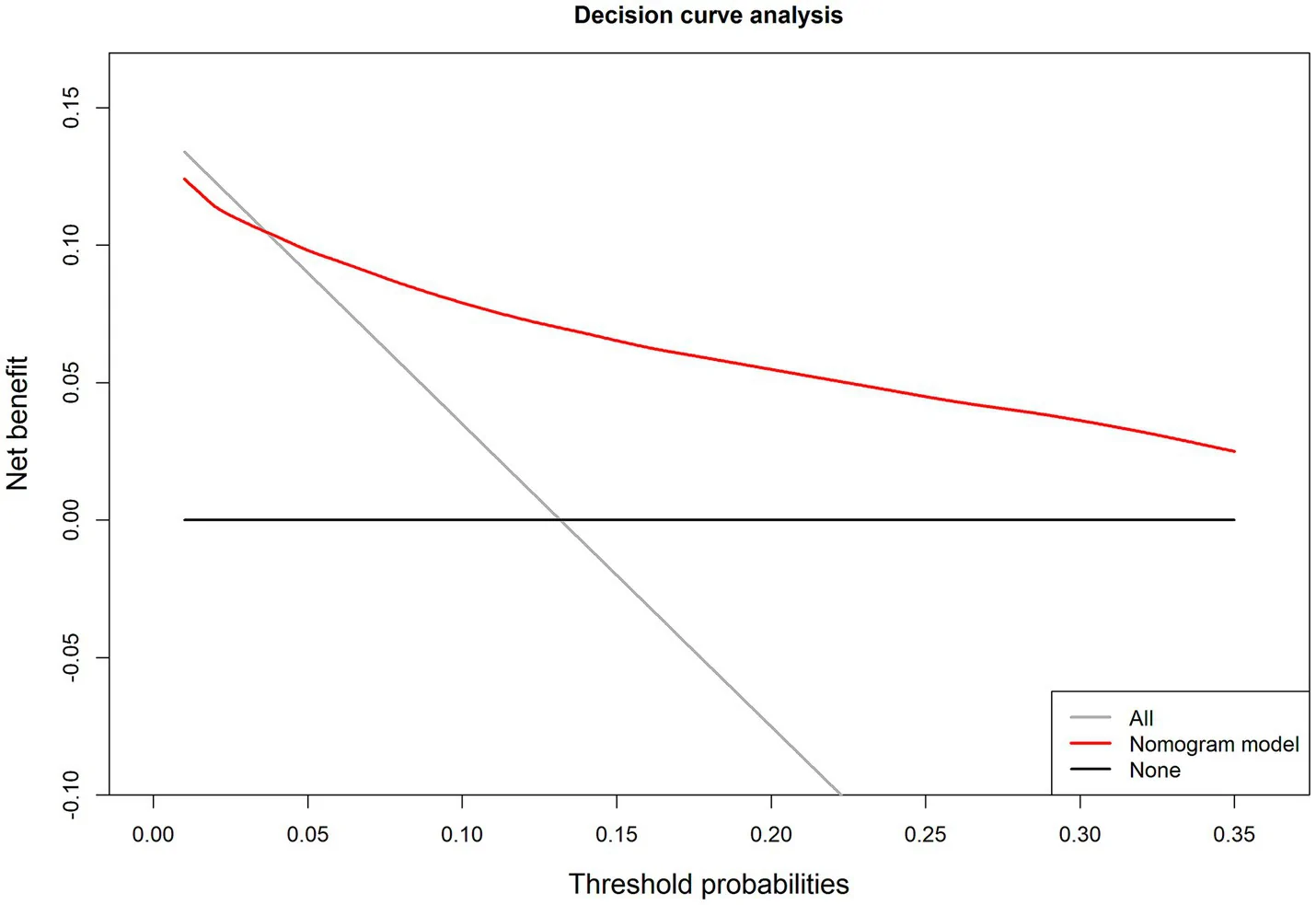
Decision curve analysis of the nomogram model for predicting chronic cough following acute bronchitis in children.

Finally, we evaluated the performance of the nomogram model by plotting the ROC curve. As shown in Figure 2A and Table 6, the AUC of the constructed prediction model was 0.819 (95% CI: 0.735–0.903), indicating excellent discrimination. Furthermore, as shown in Figure 2B, the Hosmer-Lemeshow goodness-of-fit test demonstrated that the nomogram model exhibited a good fit with favorable calibration (*p* = 0.218). As shown in Figure 3, the decision curve analysis demonstrated that within the threshold probability range of 0.03 to 0.35, the net benefit of using the Nomogram prediction model to assist decision-making was significantly superior to the two extreme strategies of ‘treat all’ and ‘treat none’. As shown in Table 6, the Nomogram model demonstrated more excellent predictive performance than the Random Forest model.

**Table 6:** Model performance metrics comparison.

Metrics	Nomogram model	Random forest model
AUC value	0.819	0.801
Accuracy (%)	83.4	82.9
Sensitivity (%)	85.7	84.9
Specificity (%)	81.5	81.2
Positive predictive value (%)	38.0	37.4
Negative predictive value (%)	97.7	97.6
Youden index	0.672	0.661

AUC: Area under the curve.

## 4 Discussion

Acute bronchitis is a common lower respiratory disease primarily caused by viral infections (2). In this study, we compared the clinical characteristics of children with acute bronchitis across different age groups and identified risk factors for the development of chronic cough, which persisted even after the resolution of other symptoms. Our findings revealed significant differences in laboratory results, such as WBC and neutrophil (Neu) counts, as well as clinical features, including fever, nasal congestion, cough, fever duration, peak temperature, and hospitalization length, across age groups. Additionally, we identified lower gestational age at birth, anemia, and parental smoking as factors independently associated with chronic cough in these children.

Previous studies have indicated that hospitalization was not necessary for acute bronchitis (2). However, recent studies indicated a rising trend in the number of children hospitalized for this condition. Approximately 5% of children admitted for viral respiratory infections were diagnosed with bronchitis (4). Furthermore, increased airborne concentrations of PM2.5, PM10, or sulfur dioxide have been linked to a significant rise in hospitalization rates for children with acute bronchitis, with children under 2 years of age being particularly vulnerable (3). In this study, our findings showed that the median length of hospitalization for children of all ages in this study was 4 days, which was similar with the previous study (4).

Respiratory diseases exhibit more pronounced gender differences, with previous studies indicating that males have higher morbidity and mortality rates from lower respiratory tract infections (1). Additionally, hospitalized children with acute bronchitis and acute bronchiolitis are more frequently male than female (4, 14). In the present study, we also observed a higher proportion of males than females among children of all ages. While gender differences in adult males may be linked to factors such as tobacco use, this does not directly apply to children (1). We hypothesize that the observed gender difference in children may be related to variations in hormone levels, immune system responses, and airway anatomy between males and females (15–17).

A significant upregulation of CRP has been reported in patients with influenza A and B viruses (18), while WBC counts were notably lower in children with influenza compared to healthy controls (19). In the present study, we observed that some children with acute bronchitis exhibited elevated CRP and decreased WBC levels. Previous studies have shown that the Neu to Lym ratio is significantly higher in children with influenza and bronchiolitis compared to uninfected children (19, 20). However, these indicators were not found to be useful in determining the severity of bronchiolitis episodes (21). In our study, we found elevated Neu levels in many children, but no significant changes in Lym levels. Additionally, it has been reported that children with COVID-19 under 1 year of age had a lower percentage of CRP and Neu increases, but a higher percentage of WBC decreases compared to older children (7). Consistent with this, our study also found that the youngest group of children with acute bronchitis had the highest percentage of WBC decreases and a lower percentage of Neu increases.

Fever, sputum production, and nasal congestion are common symptoms of respiratory viral infections (2). Previous studies have shown that older children with COVID-19 exhibit higher rates of sputum production, higher peak temperatures, and longer fever duration (7). In this study, we found that children under 1 year of age had higher rates of nasal congestion and sputum production than older children, whereas children aged ≥3 years had a higher prevalence of fever and higher peak temperatures.

Chronic cough is defined as a cough lasting >8 weeks in adults and >4 weeks in children (12). Previous studies have shown that damage to the mucous membranes of the trachea and bronchi from respiratory infections can lead to subacute cough and even the development of chronic cough (22). Approximately 34–50% of children with capillary bronchiolitis develop airway hyperresponsiveness (23), and between 20 and 40% of chronic coughs in children are attributed to post-infectious cough (PIC) following respiratory infections (9, 24, 25). This chronic cough may result from cough hypersensitivity caused by neurostimulation and neuroinflammation following viral infections (26). Chronic coughing can lead to long-term discomfort, affecting daily life, work, sleep, and overall mental health, as well as causing fatigue, poor concentration, and strained social relationships (10). Additionally, chronic cough can exacerbate other conditions, increasing the risk of disease progression and complications (27). For these reasons, we categorized chronic cough as a poor prognosis in this study. We found that lower gestational age, parental smoking, and anemia in children were independently associated with chronic cough following acute bronchitis, and these factors were used to construct a predictive model.

Prematurity and low birth weight were strongly associated with the development and progression of many respiratory diseases (28). Lower gestational age has also been identified as an independent predictor of long-term cough in children with respiratory conditions (25). Similarly, in this study, we found that lower gestational age was a risk factor for chronic cough following acute bronchitis. We hypothesize that this may be due to the underdeveloped respiratory system in preterm infants, which results in greater airway responsiveness to certain external stimuli compared to term infants (28, 29).

Tobacco use and secondhand smoke were well-established factors affecting respiratory health, with tobacco smoke pollution being a significant contributor to deaths from lower respiratory infections (1, 30). Parental and self-smoking were also key causative factors for acute bronchitis in First Nations children in Canada (31). Additionally, exposure to secondhand smoke was identified as an independent risk factor for chronic cough in nonsmokers (32). In this study, we found that parental smoking was an independent risk factor for chronic cough in children with acute bronchitis.

Malnutrition increased the risk of developing common childhood respiratory diseases and adversely affected disease prognosis. It was also an important predictor of morbidity and mortality in bronchiectasis (33). In this study, anemia was independently associated with chronic cough in children with acute bronchitis. Previous studies in adults have reported associations between iron-deficiency anemia and chronic cough or airway hyperresponsiveness, and a case report suggested potential improvement following iron supplementation (34, 35). However, these findings were derived from adult populations or individual cases and therefore cannot be directly extrapolated to children or interpreted as evidence of a causal relationship. Further pediatric studies are needed to clarify the potential role of anemia and iron status in chronic cough following acute bronchitis.

In conclusion, this study revealed significant variation in the clinical features and symptoms of acute bronchitis among children of different ages. Additionally, children with lower gestational age, anemia, and parental smoking were identified as being at higher risk for developing chronic cough. Our findings offer valuable insights into the prevention of poor prognosis in children with acute bronchitis.

There are some limitations that need to be addressed in future research. First, the sample size in this study was relatively small, and the retrospective, single-center design may limit the generalizability of our findings. Second, pathogen identification was not available, and thus subgroup analysis of acute bronchitis caused by different viral pathogens could not be performed. Third, because only hospitalized children were included, selection bias cannot be excluded, and our findings may not be generalizable to the broader pediatric population with acute bronchitis, particularly those managed in outpatient settings. Moreover, children with favorable outcomes may have been less likely to return for follow-up, potentially leading to an overestimation of chronic cough and influencing the predictive model. In addition, we defined ‘poor prognosis’ specifically as the development of chronic cough lasting longer than 4 weeks. We acknowledge that prognosis following acute bronchitis could theoretically include other endpoints such as respiratory failure, readmission, or mortality. However, acute bronchitis is primarily a self-limiting disease, and severe complications or mortality are rare in children without severe comorbidities. Therefore, these outcomes were not suitable for the prognostic analysis in our cohort. Finally, several potentially relevant confounding factors, including allergic diseases, recurrent respiratory infections, viral etiology, breastfeeding history, vaccination status, bronchopulmonary dysplasia, air pollution exposure, and medications administered around the time chronic cough was assessed, were not included in the multivariable analysis, which may have resulted in residual confounding. Although children with a prior diagnosis of asthma were excluded, the potential influence of undiagnosed or subsequent asthma could not be fully assessed. Furthermore, given the retrospective study design, the observed associations should not be interpreted as causal relationships. The prediction model was not externally validated, and the relatively small number of events may have introduced a risk of model overfitting. Therefore, the predictive performance and generalizability of the model should be interpreted cautiously and evaluated in larger, multicenter cohorts with external validation.

## Data Availability

The original contributions presented in the study are included in the article/Supplementary material, further inquiries can be directed to the corresponding author.
