# Supplementary material for: Clinical characteristics and prognostic differences of children with acute bronchitis across different age groups
Source: Front Med (Lausanne). 2026 Aug 17;13:1922135. doi: 10.3389/fmed.2026.1922135 (PMC13527008; doi:10.3389/fmed.2026.1922135)
Supplement: Supplementary file 1 [file Data_Sheet_1.DOCX]

Supplementary Material

## Supplementary Figures

**Figure S1.** Flowchart of Participant Selection and Study Population.
